# Wide Range Simulation Study of Taylor Bubbles in Circular Milli and Microchannels

**DOI:** 10.3390/mi8050154

**Published:** 2017-05-12

**Authors:** Luis A. M. Rocha, João M. Miranda, Joao B. L. M. Campos

**Affiliations:** Centro de Estudos de Fenómenos de Transporte, Departamento de Engenharia Química, Faculdade de Engenharia da Universidade do Porto, Rua Dr. Roberto Frias, 4200-465 Porto, Portugal; lrocha@fe.up.pt (L.A.M.R.); jmc@fe.up.pt (J.B.L.M.C.)

**Keywords:** Taylor bubble, gas-liquid slug flow, microchannels, millichannels, Newtonian liquids

## Abstract

A deep knowledge of the hydrodynamics of two-phase flow in millichannels and microchannels is relevant to the design and control of micro structured equipment. While there is plenty of work published in this area, there is a lack of studies over a wide range of dimensionless numbers and some factors have not been properly addressed, such as the role of the Reynolds number, the features of recirculation regions in the liquid slug and the liquid film development length. Therefore, a wide range parametric study of isolated gas Taylor bubbles flowing in co-current with liquid in circular milli- and microchannels is presented, in a wide range of Capillary (Ca_B_) (0.01–2) and Reynolds numbers (Re_B_) (0.01–700). The shape and velocity of the bubbles are, together with the flow patterns in the flowing liquid, analyzed and compared with numerical and experimental correlations available in the literature. For low values of Ca_B_, the streamlines (moving reference frame (MRF)) in the liquid slug show semi-infinite recirculations occupying a large portion of the cross-section of the channel. The mean velocity of the fluid moving inside the external envelope of the semi-infinite streamlines is equal to the bubble velocity. For high values of Ca_B_, there are no recirculations and the bubble is moving faster or at least at the velocity of the liquid in the center of the tube; this flow pattern is often called bypass flow. The results also indicate that the liquid film surrounding the bubbles is for low Ca_B_ and Re_B_ numbers almost stagnant, and its thickness accurately estimated with existing correlations. The stagnant film hypothesis developed provides an accurate approach to estimate the velocity of the bubble, in particular for low values of Ca_B_. The asymptotic behavior of the studied parameters enables the extrapolation of data for Ca_B_ lower than 0.01. In addition to the simulations of isolated bubbles, simulations with two consecutive bubbles were also carried out; coalescence was only observed in very specific conditions. The results obtained in this study are directly applicable to co-current slug flow in milli- and microchannels for 0.1 < Re_B_ < 1000 and 0.02 < Ca_B_ < 2.

## 1. Introduction

A rigorous understanding of the hydrodynamic features of milli- and micro-scale two-phase flow is of great importance to the design and control of several equipment; one example are structured reactors [1,2]. These reactors allow enhanced heat and mass transfer due to their larger surface-to-volume ratio. In two-phase flows, the most important and commonly observed flow pattern is slug flow [3]. This regime is typified by a sequence of bullet shaped gas bubbles separated by liquid slugs, with the bubbles occupying the entire channel cross-section or being at most separated from the channel wall by a thin liquid film. This liquid film contributes to the reduction of the axial mixing along the liquid flow [4,5]. In addition, the recirculation vortices, which can appear in the liquid slugs, can improve the heat and mass transfer between the liquid and the wall as well as the interfacial mass transfer from the gas to the liquid [6]. The vortices observed in the liquid slugs have also a role in the flow promoted by gas embolisms. In vitro studies show that the vortices promote complex spatio-temporal variations of the red blood cell distribution along the vessel with implications for local rheology and transport processes [7].

The dynamics of milli- and micro-scale two-phase flows are significantly different from the dynamics at the macro scale, because of the fact that the interfacial forces become more prominent than the gravitational forces, which tend to be negligible. The dimensionless number relating these two forces is the Eötvös number (often also called Bond number), defined as follows:

(1) $$\mathrm{Eo}=\frac{(\rho_{L}-\rho_{G})gD^{2}}{\gamma }$$

where $\rho_{G}$ and $\rho_{L}$ are the gas and liquid densities, respectively, *g *the acceleration due to gravity, *D* the tube diameter and *γ* the surface tension.

Several different values have been suggested as the limit for Eo under which a channel can be considered “small” and interfacial forces dominate over gravitational forces: (2π)^2^ by Suo and Griffith [8] and 3.37 by Bretherton [9], estimated differently. In channels with dimensions until a few hundred microns, the Eötvös number is far below both of these limits, regardless the properties of the fluids. Therefore, the simulations carried out in the present work neglect the gravity effect.

The main dimensionless numbers that govern two-phase co-current flow in small channels are the Reynolds number and the Capillary number based either on the bubble velocity or on the mean liquid velocity. The Reynolds number compares the inertial forces with the viscous forces and is defined through the bubble velocity as follows:

(2) ReB=ρLVBDμL

where *V*_B_ is the velocity of the bubble and *μ*_L_ the viscosity of the liquid. 

The Capillary number relates the viscous forces with the interfacial forces, and is defined as follows:

(3) $${\mathrm{Ca}}_{B}=\frac{\mu_{L}V_{B}}{\gamma }\;or\;{\mathrm{Ca}}_{L}=\frac{\mu_{L}V_{L}}{\gamma }$$

Two other dimensionless numbers can be pertinent when studying two-phase flows: the Weber number, which compares inertial and interfacial forces, and the Laplace number, which is independent of external dynamics parameters such as velocity and includes only the properties of the fluids and geometric characteristics of the channel. Laplace and Weber numbers can be expressed as functions of Re_B_ and Ca_B_:

(4) WeB=ReB×CaB=ρLVB2Dγ

(5) La=ReBCaB=ρLγDμL2

The ratio between the length of the bubble and the tube radius is another dimensionless group to have in consideration. However, this group is important only when the length of the bubble is not long enough to have a stabilized liquid film flowing between the bubble and the tube wall. In the present study, only bubbles with a sufficient length to have this film stabilized will be analyzed. 

In this work, an attempt is made to clarify, through numerical simulations, the shape and velocity of isolated Taylor bubbles as well as the thickness of the liquid film flowing between the bubble and the wall in milli- and microchannels, for a wide range of Reynolds and Capillary numbers. The paper first presents a review of literature concerning the state of the art. The subsequent section outlines the computational fluid dynamics (CFD) model used to carry out the simulations. In the Results and Discussion Section, the simulation data are presented and are validated through correlations based on experiments from the literature.

## 2. State of the Art

A review of literature concerning the main parameters studied in this paper is presented in this section. More in depth article reviews about this topic are also available [2,10].

### 2.1. Liquid Film around the Taylor Bubble

Aussillous and Quere [11] carried out experiments in capillary tubes utilizing low viscosity liquids at high velocities in order to investigate the liquid film thickness around a Taylor bubble flowing in co-current. The results were compared with data from the experiments of Taylor [12] and Bretherton [9], obtained in capillaries under negligible inertial effects. An empirical fit equation expressing the film thickness as a function of Ca_B_ was proposed:

(6) $$\frac{\delta }{R}=\frac{1.34\;{\mathrm{Ca}}_{B}^{2/3}}{1+2.5\left(1.34\;{\mathrm{Ca}}_{B}^{2/3}\right)}$$

where *δ* is the film thickness and *R* the radius of the capillary.

Aussillous and Quere noted that above a certain liquid mean velocity, the film was thicker than the data presented by Taylor, this deviation being attributed to the inertia forces involved.

Serizawa et al. [13] visualized air-water flow in circular tubes of 20, 25 and 100 μm through a microscope. During slug flow at low velocities, the authors noted that small liquid droplets were sticking on the tube wall around the gas slug and this occurrence was regarded as evidence that no liquid film exists between the gas slug and the tube wall. The effect of surface wettability was then investigated and a thin liquid film appeared at high gas velocities under carefully treated clean surface conditions. 

Warnier et al. [14] described the gas hold-up and its relation to the liquid film thickness around a Taylor bubble under conditions where inertial effects are significant. In addition, a mass balance based model for Taylor bubbles with a quiescent liquid film was developed. Applying the model to experimental data, they obtained the gas hold-up, which follows Armand’s correlation [15] for significant inertial effects. This conclusion points towards a liquid film thickness independent of the bubble velocity and equal to a fixed fraction of the channel cross-section whatever the channel diameter. This conclusion is in agreement with the qualitative analysis of Aussillous and Quere [11].

Gupta et al. [16] reviewed the occurrence of wall dry-out during experimental studies and suggested a criterion to have a sufficiently fine mesh to capture film flow in numerical simulations. The study concluded that Taylor bubbles are most of the times surrounded by liquid and dry-out at the walls is only observed under very particular conditions, such as during transient mixing of gas and liquid from separate streams, flow in noncircular geometries or at very high homogeneous void fraction. Additionally, wall adhesion properties play a role only when gas and liquid are both in contact with the wall. The authors also stressed the importance of near-wall mesh resolution in numerical studies.

Han and Shikazono [17] used a laser focus displacement meter to measure the thickness of the thin liquid film in slug flow and studied the parameters that affect its formation and development. The study presents the effects of the Capillary number, Reynolds number, gas and liquid slug lengths and gravity on the liquid film thickness. They concluded that at small Capillary numbers, the inertial effects are negligible. As Capillary number increases, the inertial effects can no longer be neglected and for Reynolds numbers above 2000, liquid film thickness becomes nearly constant. According to Han and Shikazono, liquid slug length has a weak effect on the liquid film thickness, whereas it becomes undeveloped and thicker for short gas bubbles. The gravity is only important in tubes of large diameter. Finally, they proposed an empirical correlation based on Ca_B_, Re_B_ and We_B_ to predict liquid film thickness within ±15% of accuracy:

(7) δD=0.670CaB2/31+3.13CaB2/3+0.504CaB0.672ReB0.589−0.352WeB0.629

For multiphase flows in non-circular channels, several studies have reported the occurrence of partial dry-out at the walls, with the liquid flowing only on the edges of the channel [18,19,20]. This phenomenon was observed mostly at low gas flow rates and depending on the affinity between the liquid and the wall.

According to the literature, the dry-out between the bubble and the wall only happens under certain conditions. In circular geometries, this phenomenon is reported under very low gas velocities while in noncircular geometries, this effect is more common and the wettability plays an important role together with the gas velocity. In respect to the thickness of the liquid film, it depends on the interfacial and inertial forces. If inertial forces are negligible, the film thickness should depend exclusively on the Capillary number. However, if inertial forces cannot be neglected, the Capillary number no longer affects the film flow and its thickness becomes a constant fraction of the tube diameter.

### 2.2. Taylor Bubble Velocity

Liu et al. [21] studied experimentally slug flow hydrodynamics in vertical capillaries of circular and square cross sections. The authors studied the parameters affecting the bubble rise velocity with a high-speed video camera. In order to overcome the limitations of the existing models, such as the estimation of parameters that are difficult to measure, the authors presented the following correlation, which allows the calculation of the bubble velocity through the two-phase velocity and the Capillary number based on that velocity, Ca_TP_:

(8) $$\frac{V_{B}}{V_{\mathrm{TP}}}=\frac{1}{1-0.61{\mathrm{Ca}}_{\mathrm{TP}}^{0.33}}$$

where *V*_TP_ is the two-phase velocity, defined as the sum of the gas and liquid superficial velocities. 

This equation is valid for Capillary numbers in the range between 0.0002 and 0.39. The work also concluded that geometry and hydraulic diameter have little influence on the bubble rise velocity.

Abiev and Lavretsov [22] studied the hydrodynamics of slug flow in horizontal capillaries with an inner diameter of 0.92 mm. The systems studied were water–air and glycerol–air. They compared the experimental data obtained with results from existing mathematical models and presented a new equation, based on an earlier model [23], to estimate the bubble velocity as a function of Capillary number. The approach is as follows:

(9) $$\frac{V_{B}}{V_{\mathrm{TP}}}=1+d\left[1-\exp\left(-F({\mathrm{Ca}}_{B})\right)\right]$$

where *d* is a constant. F(Ca_B_) can be one of three different functions, with increasing accuracy but also with increasing complexity, presented below:

(10) $$F_{1}\left({\mathrm{Ca}}_{B}\right)=\exp\left[b+c\ln\left({\mathrm{Ca}}_{B}\right)\right]$$

(11) $$F_{2}\left({\mathrm{Ca}}_{B}\right)=\exp\left[b+c\ln\left({\mathrm{Ca}}_{B}\right)+g\ln{\left({\mathrm{Ca}}_{B}\right)}^{2}\right]$$

(12) $$F_{3}\left({\mathrm{Ca}}_{B}\right)=\exp\left[b+c\ln({\mathrm{Ca}}_{B})+g\ln{\left({\mathrm{Ca}}_{B}\right)}^{2}+h\ln{\left({\mathrm{Ca}}_{B}\right)}^{3}\right]$$

where *b*, *c*, *g* and *h* are constants.

Another approach to estimate the bubble velocity is to assume motionless liquid film between the bubble and the channel. Under this assumption, an expression relating the bubble velocity to the mean liquid velocity is obtained from a simple mass balance:

(13) $$V_{B}A_{B}+V_{\mathrm{film}}A_{\mathrm{film}}=V_{L}A_{\mathrm{MC}}$$

(14) $$V_{B}=\frac{A_{\mathrm{MC}}}{A_{B}}V_{L}-\frac{V_{\mathrm{film}}A_{\mathrm{film}}}{A_{B}}$$

where *A*_B_, *A*_film_ and *A*_MC_ are the cross section areas of the bubble, film and microchannel, respectively, and *V*_film_ is the average velocity in the film. If *V*_film_ is zero, then the equation is simplified:

(15) $$V_{B}=\frac{A_{\mathrm{MC}}}{A_{B}}V_{L}$$

Since the radius of the bubble is $R_{B}=R_{\mathrm{MC}}-\delta$, the velocity of the bubble can be determined through the thickness of the liquid film:

(16) $$V_{B}=\frac{1}{{\left(1-\frac{\delta }{R}\right)}^{2}}V_{L}$$

According to this approach, the bubble velocity is always higher than the mean liquid velocity. This approach has already been described in the literature [8,24,25].

The literature shows that the liquid velocity is the most important parameter affecting the velocity of gas slug bubble flowing in milli- and microchannels. Other parameters, such as fluid properties and geometry have also been shown to influence bubble velocity, but with a less important contribution. Various correlations and models have been proposed to predict the bubble velocity. Usually, they are based on the two-phase velocity, with some parameters depending on dimensionless numbers such as the Capillary number.

### 2.3. Flow in Liquid Slugs

Thulasidas et al. [26] studied the flow patterns in the liquid slugs during bubble-train flow inside capillaries, in a moving reference frame (MRF). High-speed video imaging and particle image velocimetry (PIV) techniques were applied to characterize the liquid flow patterns and to determine the velocity profiles along the liquid slugs. The authors found that semi-infinite vortices or a complete bypass flow are observed, depending on the Capillary number. They presented a theoretical model to predict the position and size of the vortices and the velocity profiles inside. Three important parameters that characterize the flow in a MRF were determined: the radial position of the center of the vortex, the radial position of the streamline envelope separating the vortex from the downwards flowing liquid film and the recirculating time of a particle trapped in the vortex.

Taha and Cui [27] carried out numerical simulations with the VOF method to study the hydrodynamics of slug flow in capillaries. Velocity and bubble shape were obtained as a function of the Capillary number together with a detailed description of the velocity field around the bubble. The simulations, in MRF, presented a strong vortex in the liquid slug ahead of the bubble. With increasing Capillary number, the vortex becomes narrower, radial direction, and the radial coordinate of the center shifts towards the capillary axis. The transition to bypass flow occurs, according to the authors, above Ca = 0.5. These numerical results are in agreement with previously published experimental results.

Zaloha et al. [28] studied the hydrodynamics of liquid slugs in gas-liquid Taylor flow in straight and meandering microchannels using micro particle image velocimetry (µPIV). The results are in agreement with previous studies, confirming a recirculation motion in the liquid slug (MRF), which is symmetrical about the centerline of the channel for the straight geometry and more complex and three dimensional in the meandering channel. This study also quantified the recirculation motion by evaluating the recirculation rate, velocity and time. Zaloha et al. found that the recirculation velocity increases linearly with the two-phase superficial velocity *V*_TP_ and is independent of the superficial velocity ratio *V*_G_/*V*_L_ for the studied conditions. 

Experimental and numerical studies have shown that the differences reported between the flow in circular and square capillaries are because as the Capillary number approaches zero, the liquid film thickness tends to zero in circular capillaries, but the flow in the corners remains in square capillaries. 

The purpose of this work is to provide a wide range study of isolated gas bubbles flowing in liquids in capillaries and in microchannels, through a complementary analysis of the shape and velocity of the bubbles and of the flow pattern in the liquid. In spite of the array of studies in this area, there is no systematic and inclusive study enclosing so wide range of scales. In addition, the effects of the Reynolds number are not always considered and the features of the recirculation regions in the liquid slugs reported in the literature are not well defined. These objectives will be reached through numerical simulations based on a dimensionless analysis of the phenomena in study. In addition, the dynamics of two consecutive Taylor bubbles are also analyzed to investigate the occurrence of coalescence, a topic not found in literature.

### 2.4. CFD Model

The commercial software ANSYS Fluent (Release 16.2.0, ANSYS, Inc., Canonsburg, PA, USA) was used in this study to perform the numerical simulations, following a similar approach to that used in other multiphase numerical studies [27,29]. Computational Fluid Dynamics requires a differential description of the fluid flow. The momentum equation is given by

(17) $$\frac{\partial }{\partial t}\left(\rho \vec{v}\right)+\nabla ∙\left(\rho \vec{v}\vec{v}\right)=-\nabla p+\nabla ∙\left[\mu (\nabla \vec{v}+\nabla {\vec{v}}^{T})\right]-\rho g+\vec{f}$$

where *p* is the pressure and $\vec{f}$ represents the surface tension contribution, added as a source term to the equation. The continuity equation is the following,

(18) $$\frac{\partial \rho }{\partial t}+\nabla ∙\left(\rho \vec{v}\right)=0$$

In order to model two-phase flow, the VOF method was applied, which uses a variable *α_i_* to track the position of the interface. This variable is 1 at any point exclusively occupied by fluid *i* and 0 if *i* is not present. In the interface between both fluids, the variable assumes a value between 1 and 0. Therefore,

(19) $$\alpha_{C}+\alpha_{D}=1$$

The tracking of the interface is computed with the following equation,

(20) $$\frac{\partial \alpha_{i}}{\partial t}+\left({\vec{v}}_{i}∙\nabla \right)\alpha_{i}=0$$

together with the geometric reconstruction scheme which assumes a piecewise-linear approach to represent the interface between phases [30,31].

Surface tension is modeled with the continuum surface force (CSF) model [32],

(21) $${\vec{f}}_{\sigma }=\gamma k\frac{\rho \nabla \alpha_{D}}{0.5(\rho_{C}+\rho_{D})}$$

where *k* refers to the curvature of the interface, given by

(22) $$k=\;-\nabla ∙\frac{\nabla \alpha_{D}}{\left|\vec{\nabla }\alpha_{D}\right|}$$

The density and viscosity depend on the phase composition and on the properties of each cell and are calculated by

(23) $$\rho =\rho_{C}\alpha_{C}+\rho_{D}\alpha_{D}$$

(24) $$\mu =\mu_{C}\alpha_{C}+\mu_{D}\alpha_{D}$$

As the bubble Capillary number decreases below 0.03, spurious currents tend to appear around the interface of the bubble, inducing fictitious recirculations in the liquid slug and making it difficult to apply to the flow of low viscous liquids flowing in microchannels. In order to minimize these spurious currents, the coupled VOF-level-set method with Heaviside correction was used. The simulations were carried out in a two-dimensional and axisymmetric coordinate system. The flow can be assumed to be symmetrical since the liquid flow in the cylindrical tube is laminar and asymmetries, in such conditions, have never been reported in the literature. The reference domain therefore consists of a rectangle with a length of 700 µm and a width of 50 µm, corresponding to a circular microchannel with a diameter of 100 µm. The critical factor is the number of mesh elements in the liquid film. Gupta et al. [16] demonstrated that simulations of bubbles in microchannels require at least five elements in the film region in the radial direction, otherwise the film may not be captured at all. Three different meshes were thus used depending on the thickness of the liquid film. Mesh sensitivity tests showed that the meshes selected assure an accurate solution with minimal computational effort. In Table 1, the characteristics of the used meshes, as well as the ranges for which they are applied, are presented. All meshes are uniform.

The initial bubble shape was made from a rectangle and two semicircles on each side of the rectangle, with a radius equal to the width of the rectangle. This is the most similar shape to the bubble final form that it is possible to create in ANSYS Fluent as an initial condition. The closer the initial shape is to the correct one, the faster the simulation will be. The width of the rectangle was estimated by calculating the thickness of the liquid film taking the correlation of Han and Shikazono [17]. The length of the rectangle was always fixed as 200 µm. Numerical simulations were also carried out to study the hydrodynamics of two consecutive Taylor bubbles. The CFD methodology was identical to the one used in the analysis of isolated Taylor bubbles, with the only difference being the use of a twice as long simulation domain, with two bubbles placed at a certain distance of each other at the beginning of the simulation.

**Figure 1:**
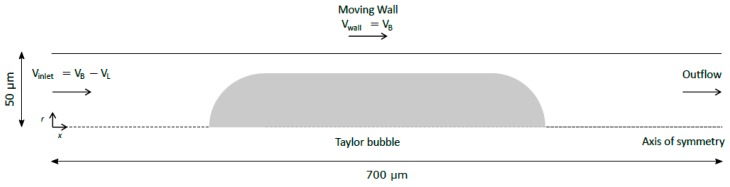
Schematic representation of the simulated domain.

Every simulation was done in a frame of reference moving with the bubble (MRF), i.e., imposing to the wall the velocity of the bubble. Since the liquid is flowing in co-current, a fully developed laminar velocity profile was imposed through a user-defined function at the inlet of the domain. At the outlet, a pressure outlet boundary condition was assumed. The velocity and pressure initial conditions were set to zero. As the simulation progressed, the velocity of the wall was iterated until the bubble nose remained stationary. As a result, the velocity of the bubble is one of the outputs of the simulation. A schematic representation of the simulated domain is present in Figure 1.

To solve the equations, the pressure-based solver was chosen which uses a finite volume approach to discretize the equations. The pressure-velocity coupling scheme used was pressure implicit with splitting of operators (PISO) and the pressure staggering option (PRESTO!) method was used for the pressure interpolations. The quadratic upstream interpolation for convective kinematics (QUICK) scheme was chosen to solve the momentum equation. The gradients of the scalars were computed with the Green–Gauss node based method.

The simulations were carried out with two cores from an Intel i5 processor, in a computer with 8 GB of random-access memory (RAM). The computational time of each simulation is dependent on the number of elements of the mesh, but most simulations were completed after 24 h.

## 3. Results and Discussion

The two main dimensionless numbers that affect gas-liquid flows in capillaries and microchannels are the Capillary number (Ca_B_ or Ca_L_) and the Reynolds number (Re_B_). Therefore, a series of simulations were carried out in the range of 0.01–2 for Ca_B_ and 0.01–700 for Re_B_. The limits of the studied Ca_B_/Re_B_ map were imposed by several restrictive factors. Below Ca_B_ = 0.01, spurious currents affect significantly the numerical solution while Ca_B_ > 2 is unrealistic for flows in milli- or microchannels. In addition, for Re_B_ higher than 700, the flow becomes 3D.

**Figure 2:**
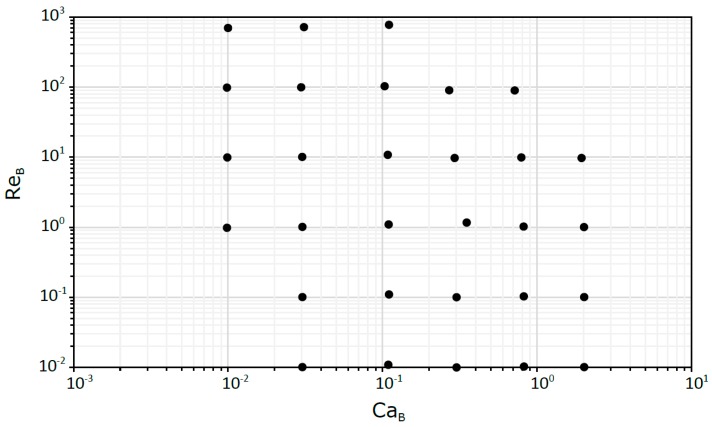
The different simulated conditions in terms of Ca_B_ and Re_B_.

The different dimensionless numbers were generated by varying the input parameters of the numerical simulations. Regarding the properties of the fluids, the density and surface tension values were maintained for all studied cases, and only the viscosity of the liquid was changed, i.e., the common property of the dimensionless numbers. The different conditions analyzed are presented in a Re_B_ versus Ca_B_ plot in Figure 2.

It is important to note that, as shown in Table 2, the dimensionless numbers that define each simulation are not always round numbers, and in some cases within a certain Ca_B_ or Re_B_ group they can have slightly different values. This is due to the CFD methodology described above, which involved iterating the bubble velocity. However, as a matter of convenience, the results presented in the next sections will neglect these differences.

**Figure 3:**
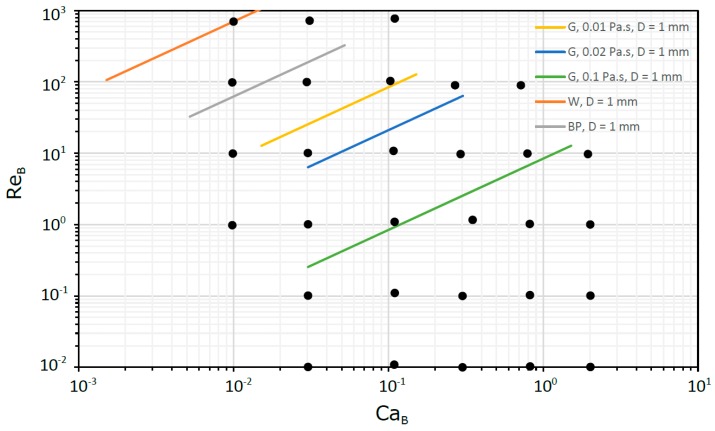
Flows in circular tubes in the milli scale.

**Figure 4:**
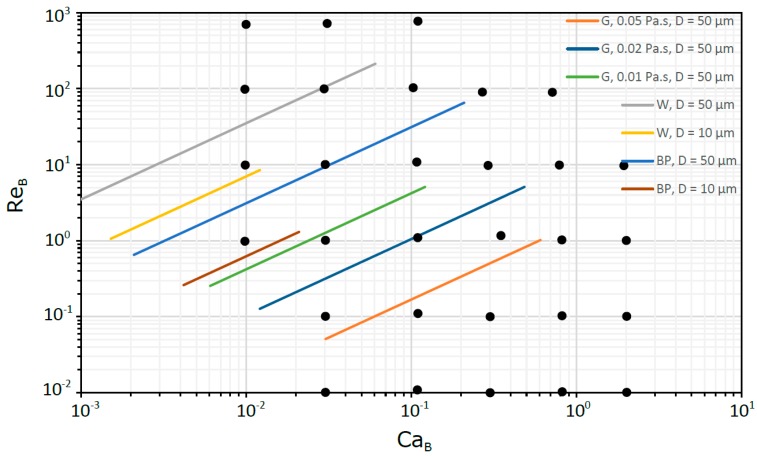
Flows in circular tubes in the micro scale.

This approach, based on dimensionless numbers, allows us to get knowledge in a wide range of practical situations ranging from milli to micro scale. In Figure 3 and Figure 4, for milli and micro scales, respectively, are located some flows in circular tubes to which the dimensionless study is suitable. The fluids selected were water (W), blood plasma (BP) and glycerol solutions (G). For the capillary tubes, the pressure drop was always less than 10^5^ Pa in 100 mm of tube length, while for the micro tubes was less than 4 × 10^5^ Pa in 1 mm of tube length.

In the micro scale domain, the region for low Ca_B_ (<0.01) was not covered due to numerical shortcomings already mentioned (spurious currents). However, as shown later, the main features of the hydrodynamic parameters in this region are easily extrapolated due to their asymptotic behavior.

### 3.1. Bubble Shape

**Figure 5:**
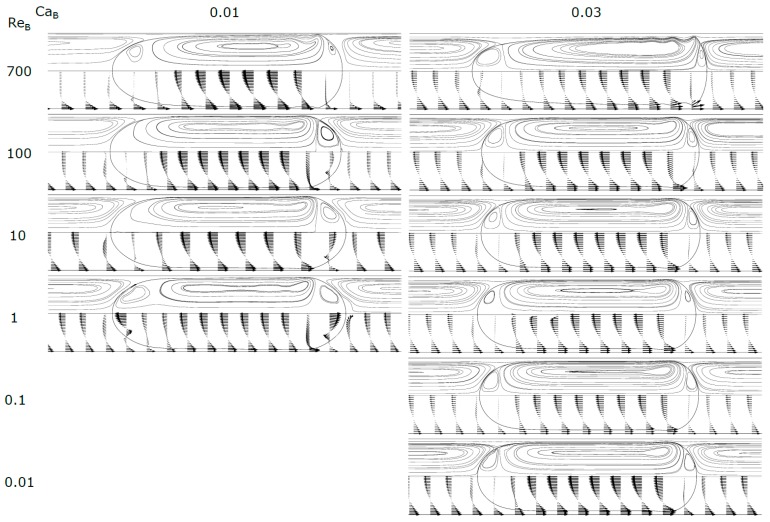
Contours of the bubble, streamlines and velocity vectors for Ca_B_ = 0.01 and 0.03.

**Figure 6:**
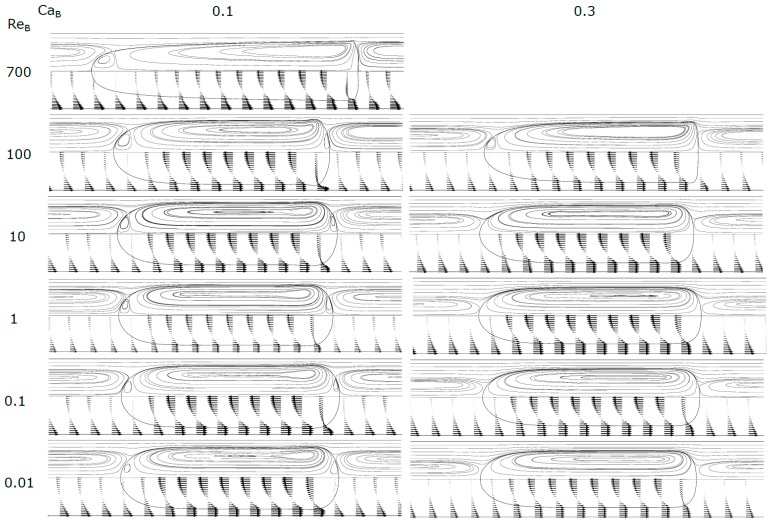
Contours of the bubble, streamlines and velocity vectors for Ca_B_ = 0.1 and 0.3.

**Figure 7:**
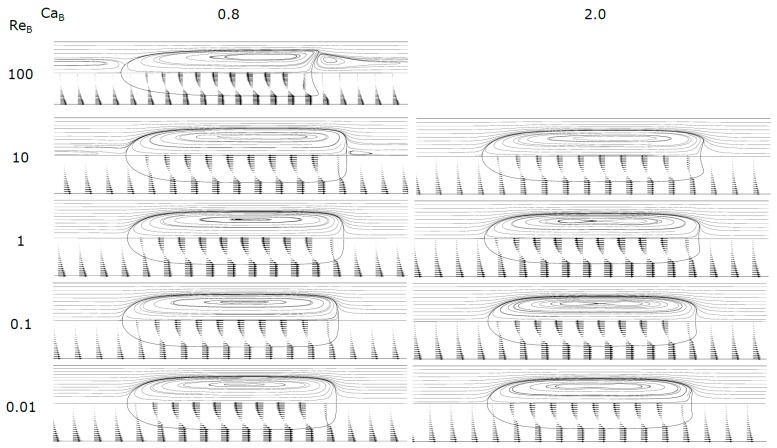
Contours of the bubble, streamlines and velocity vectors for Ca_B_ = 0.8 and 2.0.

**Figure 8:**
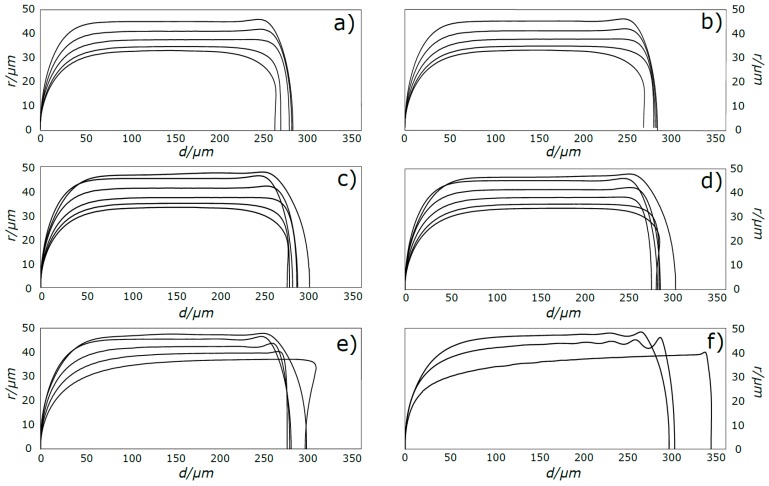
Contours of the bubble: (**a**) Re_B_ = 0.01; (**b**) Re_B_ = 0.1; (**c**) Re_B_ = 1; (**d**) Re_B_ = 10; (**e**) Re_B_ = 100; and (**f**) Re_B_ = 700. Thinner bubbles correspond to higher Ca.

Figure 5, Figure 6 and Figure 7 show the shape of the bubble, together with the streamlines and the velocity vectors in a MRF. An increase in Ca_B_ causes the front of the bubble to become slenderer for the whole range of Re_B_ studied. The rear of the bubble tends to become more concave for higher Ca_B_, being this effect more accentuated for high Re_B_. Additionally, for high Re_B_, appear some stationary wavy patterns near the back of the bubble. Figure 8 shows the contours of the bubble for each Re_B_ studied.

For values of Ca_B_ < 0.3, the streamlines (MRF) in the liquid slug show semi-infinite recirculations, occupying a larger part of the cross-section of the channel. The extent of the cross-section occupied increases as Ca_B_ decreases. The mean velocity of the fluid moving inside the external envelope of these streamlines is equal to the bubble velocity. The fluid placed ahead the bubble, between the external envelope and the tube wall, moves at a lower velocity than the bubble and will be surpassed by the bubble. As Ca_B_ decreases, the thickness of the film also decreases, and according to the stagnant film hypothesis (Equation (14)) the bubble velocity also decreases. The center of the recirculation vortex moves away from the center of the channel into the wall direction. Previous studies found in literature report similar recirculations in MRF [27], with many of them focused on the flow pattern in liquid slugs between two bubbles [26,28,33]. This topic will be developed later in the text. For Ca_B_ = 0.8, there are no recirculations and the bubble is moving faster or at the velocity of the liquid in the center of the tube; this flow pattern is often called bypass flow.

At Ca_B_ = 0.8 and Re_B_ = 100 appears, at the rear of the bubble, a recirculation region similar to that observed in vertical macro studies, significant gravity and inertial effects, in laminar regime, the so called wake region where the fluid circulates in a toroidal vortex [34]. The appearance of this region is a consequence of the expansion of the liquid film at the rear of the bubble. 

### 3.2. Liquid Film

**Figure 9:**
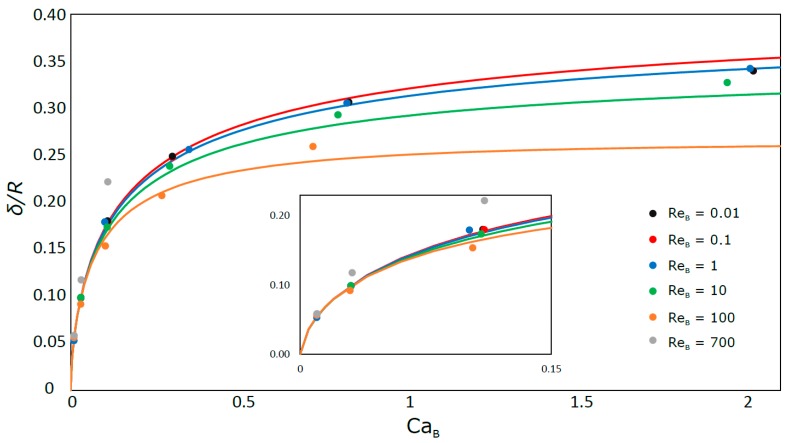
Liquid film thickness as a function of Ca_B_. The solid lines represent the correlation of Han and Shikazono [17].

Figure 9 shows the liquid film thickness as a function of Re_B_ and Ca_B_. The close symbols refer to the simulated cases and the solid lines represent the correlation of Han and Shikazono [17], which predicts the liquid film thickness with ±15% accuracy. As previously referred the film thickness decreases for decreasing Ca_B_. According to the figure, for values of Ca_B_ lower than 0.2 the liquid film thickness is independent of Re_B_. For values of Ca_B_ lower than 0.01, region not covered in the simulations, the liquid film thickness tends asymptotically to zero, i.e., the bubble tends to flow occupying all the cross-section of the tube like a two-phase plug flow.

The simulation data are in quite good agreement with the experimental correlation results. The deviation is usually below 5%, surpassing that error in three occasions: 8% in two simulations at the lowest Ca_B_, where it is harder to simulate the liquid film; for the highest bubble velocity (Ca_B_ = 0.110; Re_B_ = 772.7) where the error is about 23%. This large deviation is probably because under those conditions the liquid film is not yet fully stabilized for that bubble length.

**Figure 10:**
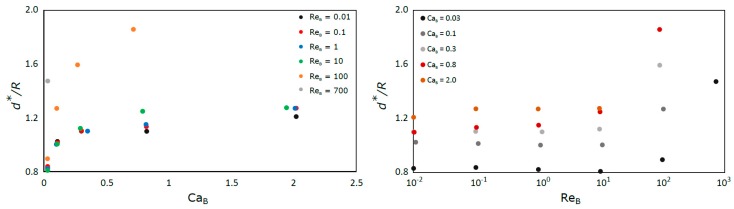
Liquid film development length as a function of: Ca_B_ (**left**); and Re_B_ (**right**).

The development length of the liquid film is defined as the distance from the bubble nose after which the film assumes a constant thickness and the velocity profile in the liquid becomes constant. This characteristic of Taylor bubbles has been studied at the macro scale [35,36], where the effects of gravity are significant, but no information was found in literature for bubbles in capillaries and microchannels. To determine this length, the derivative of the contour of the bubble was determined as a function of the distance from the front of the bubble. According to the criteria stated, the development length is reached when the average of five consecutive points of the derivative function is below 0.01. The results obtained are presented in Figure 10.

The results show that the normalized development length (d*/*R*) increases with the Capillary number, but until Re_B_ = 10 it is almost independent of Reynolds number. For Re_B_ = 100 there is a notable increase of this length for all the Capillary numbers. 

The results for Ca_B_ = 0.01 are not presented in Figure 10 since these conditions were simulated with the coupled VOF-level-set method with Heaviside correction to reduce spurious currents. While the solutions obtained with this method are still in agreement with the presented correlations for bubble velocity and liquid film thickness, the method causes the nose and back of the bubbles to have a higher radius of curvature than expected, (this effect is particularly evident in Figure 8). Consequently, the parameter d* is abnormally high in those cases.

For low values of Ca_B_ the values of the development length tend to be very small since the liquid film tends to disappear. Further work is necessary to have a better understanding of the behavior of this parameter for Re_B_ higher than 10

### 3.3. Bubble Velocity

**Figure 11:**
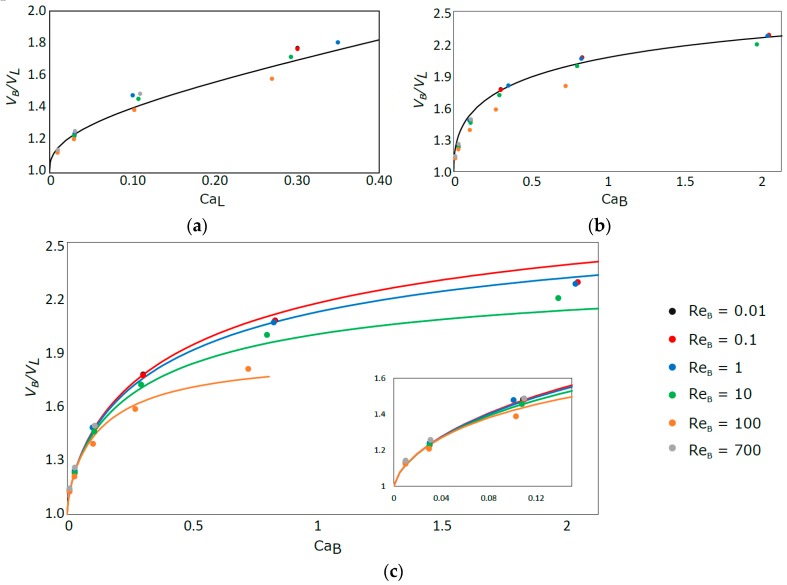
Comparison of the experimental results of $V_{B}/V_{L}$ vs. Ca_L_ with the correlation of: Liu et al. [21] (**a**); Abiev and Lavretsov [22] (**b**); and stagnant film hypothesis correlation, calculated for different Re_B_ (**c**).

In Figure 11, the simulation results for the ratio between the bubble velocity and the liquid velocity and their comparison with existing correlations are presented. It is observed that the correlation of Liu et al. [21] is in very good agreement with the simulation data for high Reynolds numbers. However, an increase of the deviation is observed with decreasing Re_B_, up to a maximum around 12% for the lowest Re_B_. This is mainly because this correlation is based on experiments performed with a range of higher Reynolds numbers than the ones used in this work. It is important to note that this correlation is a function of Ca_L_ and not Ca_B_ like the others.

The data from Abiev and Lavretsov [22] are closer to the simulation results for lower Re_B_, with the deviation increasing for increasing Re_B_ until a maximum of 10%. This behavior is due to the fact that the model equation is based on the correlation of Aussillous and Quere [11], valid mostly for low Ca_B_ and Re_B_, to estimate the thickness of the liquid film [23].

The ratio between the bubble velocity and the liquid velocity was also compared with the stagnant film correlation (Equation (16)). The solid lines (Figure 11c) correspond to the ratio calculated under that hypothesis, with the thickness of the film determined with the correlation of Han and Shikazono [17] (Equation (7)). The velocity of the bubble is shown to be in very good agreement with the stagnant film hypothesis, in particular for low values of Ca_B_.

In the low Ca_B_ region, not covered in the numerical simulations, the bubble velocity tend to the mean liquid velocity and, as already referred, the bubble tend to flow as a plug integrated inside the liquid.

These results also illustrate the fact that the bubble always travels faster than the average velocity of the liquid and can even flow faster than the maximum velocity in the liquid. Assuming the stagnant film hypothesis and relating the liquid film thickness to Ca_B_ number through the correlation of Aussillous and Quere [11], it is possible to roughly estimate the value of the Capillary number above which the bubble moves faster than the liquid in the center of the channel. Since for laminar flow the liquid in the center of the channel is flowing twice as fast as the average liquid velocity, the intended Capillary number, according to Figure 9, is around 0.74. This value should only serve as a rough estimate, mostly valid for low Re_B_ (until Re_B_ = 1).

**Figure 12:**
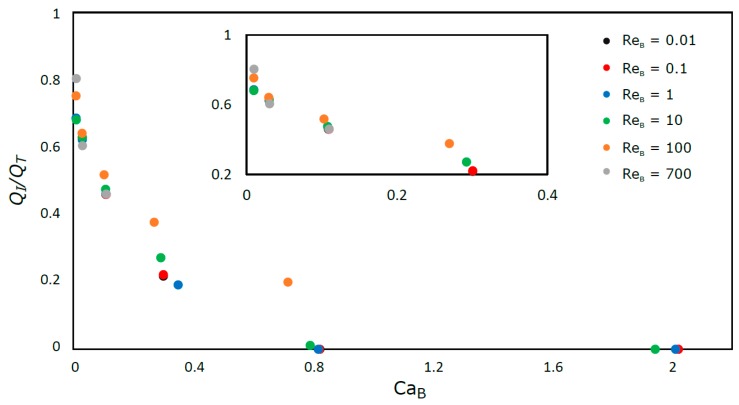
Ratio between the flow of liquid moving faster than the bubble and the total liquid flow against Ca_B_.

In Figure 12, the ratio between the liquid flow rate that is not bypassed by the bubble (*Q*_I_), i.e., with a mean velocity equal to the bubble, and the total liquid flow rate (*Q*_T_) is plotted versus Ca_B_.

For Re_B_ ≤ 10, whatever the Ca_B_ number, the curve is unique. All the fluid moves slower than the bubble for Ca_B_ ≥ 0.8. For Re_B_ = 100 and Ca_B_ = 0.8, the appearance of a toroidal closed recirculation region at the rear of the bubble (Figure 7) changes this behavior. This wake region travels at the bubble velocity and slows down the expansion of the liquid film at the rear of the bubble. This slow deceleration implies an acceleration of the liquid, in the flow direction, in front of the bubble.

The results in Figure 12 also illustrate the fact that, for very low Capillary numbers, the bubble does not bypass any liquid, moving as a plug.

### 3.4. Simulations with Two Consecutive Bubbles

When a bubble is flowing ahead or behind another bubble, the simulations showed that the main flow characteristics remain generally unchanged when compared to those of isolated bubbles flowing under the same dimensionless numbers. For the range of Re_B_ and Ca_B_ studied, coalescence was only observed once, when Reynolds and Capillary numbers are simultaneously high (Ca_B_ = 0.8; Re_B_ = 100). The coalescence only happened for the shortest distance tested ( $d_{B}$ = 61 μm). This pair of Re_B_ and Ca_B_ corresponds to the appearance of the closed recirculation wake at the rear of the bubble (Figure 7). This wake influences the reattachment of the velocity profile at the rear of the bubble and induces, near the center of the tube, regions with increased velocity. These regions are responsible by the accelerating of the following bubble.

**Figure 13:**
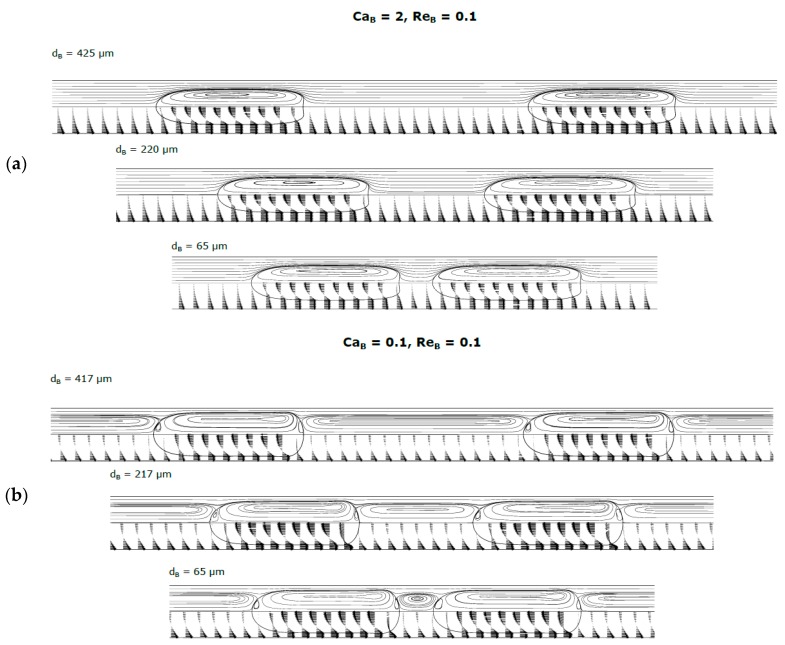
Contours of the bubbles, streamlines and velocity vectors for two consecutive bubbles. (**a**) Ca_B_ = 2; Re_B_ = 0.1; (**b**) Ca_B_ = 0.1; Re_B_ = 0.1

Two illustrative cases without coalescence are presented in Figure 13: one where recirculation vortices are observed in the liquid in front of the bubble (Ca_B_ = 0.1; Re_B_ = 0.1) and another where no recirculation vortices are present (Ca_B_ = 2; Re_B_ = 0.1). The shape, thickness of the liquid film and velocity of the bubbles are identical to those for isolated bubbles, whatever the distance between the bubbles. As for the flow of an isolated bubble, the liquid for high Ca_B_ is under “bypass flow” while for low Ca_B_ the recirculation vortices between the bubbles are, as expected, no longer semi-infinite, forming a closed vortex.

## 4. Conclusions

In this work, 2D numerical simulations of isolated and consecutive bubbles in circular milli- and microchannels were performed to study their shape, velocity, liquid film around the bubble and flow patterns in the liquid. The simulations were carried out in the range of 0.01–2 for Ca_B_ and of 0.01–700 for Re_B_ and the results are in good agreement with correlations and experimental results found in the literature. Outside of this range, a different approach is necessary, as spurious currents become more important for Ca_B_ < 0.01 and 3D effects start to appear at higher Re_B_. However, for Ca_B_ < 0.01, the results are easily extrapolated. For high Reynolds numbers and Ca_B_ > 0.8, a recirculation region at the rear of the bubble appears, as in vertical slug macro studies.

The front and rear of the bubbles are rounder at lower Capillary numbers, with the front of the bubble becoming slenderer and the rear more concave with increasing Ca_B_ and Re_B_. The velocity of the bubble, for low values of Ca_B_, is consistent with the stagnant film hypothesis, implicating that the bubble travels in the limit at the mean velocity of the liquid. 

In a moving reference frame, the fact that some parts of the liquid are moving faster than the bubble cause the appearance of semi-infinite recirculation regions in the liquid in front and behind the bubble. At higher Ca_B_, the bubble moves at a velocity higher than the highest velocity on the liquid, and no recirculation regions appear. 

The thickness of the liquid film around the bubble is accurately predicted with existing correlations, but there is still a lack of information regarding the development length of the film for high Reynolds number. The results suggest that d* increases with Ca_B_ until a plateau is reached, with its value being higher for higher Re_B_.

Regarding the velocity of the bubble, the stagnant film hypothesis was shown to be an accurate approach for low values of Ca_B_. If the thickness of the liquid film is unknown, other correlations are available, but these do not account with the effects of the Reynolds number. The correlations of Liu et al. [21] and Abiev and Lavretsov [22] were found to be more accurate for higher and lower Re_B_, respectively.

The main parameters studied remained generally unchanged when analyzed in consecutive bubbles, and coalescence was only observed in particular conditions of Re_B_ and Ca_B_ (Ca_B_ = 0.8; Re_B_ = 100).

The results obtained in this study are directly applicable to co-current slug flow in milli- and microchannels for 0.1 < Re_B_ < 1000 and 0.02 < Ca_B_ < 2.

## Figures and Tables

**Table 1:** Meshes used in this work.

Mesh	Ca Range	Number of Elements	Size of the Elements
1	≥0.1	35,000	1 × 1 µm^2^
2	>0.01	140,000	0.5 × 0.5 µm^2^
3	=0.01	140,000	0.25 × 1 µm^2^

**Table 2:** Parameters for each simulated case.

Ca_B_	Re_B_	*V*_L_ (m/s)	*V*_B_ (m/s)	*V*_B_ */V*_L_	*δ/R*
0.00983	0.982	0.0730	0.0822	1.13	0.0532
0.00990	9.90	0.231	0.262	1.14	0.0582
0.00983	98.2	0.730	0.822	1.13	0.0564
0.0100	699	1.93	2.21	1.14	0.0584
0.0303	0.0101	0.0119	0.0146	1.23	0.0988
0.0303	0.101	0.0376	0.0462	1.23	0.0984
0.0303	1.02	0.119	0.147	1.24	0.0982
0.0302	10.1	0.376	0.462	1.23	0.0988
0.0297	99.2	1.19	1.44	1.21	0.0918
0.0310	721	3.15	3.96	1.26	0.118
0.109	0.0109	0.0195	0.0290	1.48	0.180
0.110	0.110	0.0620	0.0919	1.48	0.180
0.101	1.10	0.196	0.290	1.48	0.179
0.108	10.8	0.620	0.904	1.46	0.174
0.103	103	1.96	2.73	1.39	0.154
0.110	773	5.19	7.73	1.49	0.222
0.301	0.0100	0.0260	0.0460	1.77	0.249
0.301	0.100	0.0823	0.145	1.77	0.248
0.350	1.17	0.296	0.534	1.81	0.256
0.293	9.76	0.824	1.41	1.72	0.238
0.270	89.9	2.61	4.12	1.58	0.207
0.823	0.0103	0.0372	0.0770	2.07	0.306
0.821	0.103	0.118	0.243	2.07	0.307
0.818	1.02	0.372	0.766	2.06	0.305
0.791	9.89	1.18	2.34	1.99	0.293
0.717	89.6	3.72	6.70	1.80	0.259
2.02	0.0101	0.0524	0.120	2.28	0.340
2.02	0.101	0.166	0.378	2.28	0.340
2.01	1.01	0.524	1.19	2.27	0.342
1.94	9.71	1.66	3.63	2.19	0.327

## Nomenclature

$A_{B}$	Cross section area of the bubble	m^2^
$A_{\mathrm{film}}$	Cross section area of the liquid film	m^2^
$A_{\mathrm{MC}}$	Cross section area of the microchannel	m^2^
*D*	Internal diameter of the tube	m
$d^{*}$	Liquid film development length	m
$d_{B}$	Distance between two consecutive bubbles	m
$\vec{f}$	Surface tension contribution	Pa·m^−1^
*g*	Acceleration due to gravity	m·s^−2^
*k*	Curvature of the interface	m^−1^
$L_{s}$	Bubble length	m
*p*	Pressure	Pa
$Q_{I}$	Volumetric flow rate of the liquid moving faster than the bubble	m^3^·s^−1^
$Q_{T}$	Volumetric flow rate of the liquid flowing in the tube	m^3^·s^−1^
*R*	Internal radius of the tube	m
$\vec{v}$	Velocity vector	m·s^−1^
$V_{B}$	Taylor bubble velocity	m·s^−1^
$V_{\mathrm{film}}$	Mean velocity in the liquid film	m·s^−1^
$V_{\mathrm{inlet}}$	Mean velocity at the inlet	m·s^−1^
$V_{L}$	Mean velocity in the liquid	m·s^−1^
$V_{\mathrm{TP}}$	Two-phase velocity	m·s^−1^
$V_{\mathrm{wall}}$	Velocity of the wall in the CFD model	m·s^−1^
*x,r*	Coordinates in the 2D simulation domain	m

## Greek Letters

$\alpha_{i}$	Phase indicator (phase *i*)	
$\alpha_{C}$	Phase indicator (continuous phase)	
$\alpha_{D}$	Phase indicator (dispersed phase)	
γ	Surface tension	N·m^−1^
δ	Annular liquid film thickness	m
*μ*	Viscosity	Pa·s
$\mu_{C}$	Viscosity of the continuous phase	Pa·s
$\mu_{D}$	Viscosity of the dispersed phase	Pa·s
$\mu_{L}$	Viscosity of the liquid	Pa·s
*ρ*	Density	kg·m^3^
$\rho_{C}$	Density of the continuous phase	kg·m^3^
$\rho_{D}$	Density of the dispersed phase	kg·m^3^
$\rho_{L}$	Density of the liquid	kg·m^3^
$\rho_{G}$	Density of the gas	kg·m^3^

## Dimensionless Groups

${\mathrm{Ca}}_{B}$	Capillary number based on the velocity of the Taylor bubble
${\mathrm{Ca}}_{L}$	Capillary number based on the mean velocity of the liquid
Eo	Eötvös number
La	Laplace number
ReB	Reynolds number based on the velocity of the Taylor bubble
ReL	Reynolds number based on the mean velocity of the liquid
${\mathrm{We}}_{B}$	Weber number based on the velocity of the Taylor bubble

## List of Acronyms

CFD	Computational fluid dynamics
FFR	Fixed frame of reference
MFR	Moving frame of reference
PIV	Particle image velocimetry
VOF	Volume-of-fluid
